# Whole exome sequence-based association analyses of plasma amyloid-β in African and European Americans; the Atherosclerosis Risk in Communities-Neurocognitive Study

**DOI:** 10.1371/journal.pone.0180046

**Published:** 2017-07-13

**Authors:** Jeannette Simino, Zhiying Wang, Jan Bressler, Vincent Chouraki, Qiong Yang, Steven G. Younkin, Sudha Seshadri, Myriam Fornage, Eric Boerwinkle, Thomas H. Mosley

**Affiliations:** 1 Gertrude C. Ford MIND Center, University of Mississippi Medical Center, Jackson, Mississippi, United States of America; 2 Department of Data Science, John D. Bower School of Population Health, University of Mississippi Medical Center, Jackson, Mississippi, United States of America; 3 Human Genetics Center, Department of Epidemiology, Human Genetics & Environmental Sciences, School of Public Health, University of Texas Health Science Center, Houston, Texas, United States of America; 4 Lille University, Inserm, CHU Lille, Institut Pasteur de Lille, U1167 - RID-AGE - Risk factors and molecular determinants of aging-related diseases; Lille, France; 5 Department of Biostatistics, Boston University School of Public Health, Boston, Massachusetts, United States of America; 6 The National Heart, Lung, and Blood Institute’s Framingham Heart Study, Framingham, Massachusetts, United States of America; 7 Department of Neuroscience, Mayo Clinic College of Medicine, Mayo Clinic Jacksonville, Jacksonville, Florida, United States of America; 8 Department of Neurology, Boston University School of Medicine, Boston, Massachusetts, United States of America; 9 The Brown Foundation Institute of Molecular Medicine, Research Center for Human Genetics, The University of Texas Health Science Center, Houston, Texas, United States of America; 10 Human Genome Sequencing Center, Baylor College of Medicine, Houston, Texas, United States of America; 11 Department of Medicine, University of Mississippi Medical Center, Jackson, Massachusetts, United States of America; Universiteit Antwerpen, BELGIUM

## Abstract

**Objective:**

We performed single-variant and gene-based association analyses of plasma amyloid-β (aβ) concentrations using whole exome sequence from 1,414 African and European Americans. Our goal was to identify genes that influence plasma aβ_42_ concentrations and aβ_42_:aβ_40_ ratios in late middle age (mean = 59 years), old age (mean = 77 years), or change over time (mean = 18 years).

**Methods:**

Plasma aβ measures were linearly regressed onto age, gender, *APOE* ε4 carrier status, and time elapsed between visits (fold-changes only) separately by race. Following inverse normal transformation of the residuals, seqMeta was used to conduct race-specific single-variant and gene-based association tests while adjusting for population structure. Linear regression models were fit on autosomal variants with minor allele frequencies (MAF)≥1%. T5 burden and Sequence Kernel Association (SKAT) gene-based tests assessed functional variants with MAF≤5%. Cross-race fixed effects meta-analyses were Bonferroni-corrected for the number of variants or genes tested.

**Results:**

Seven genes were associated with aβ in late middle age or change over time; no associations were identified in old age. Single variants in *KLKB1* (rs3733402; p = 4.33x10^-10^) and *F12* (rs1801020; p = 3.89x10^-8^) were significantly associated with midlife aβ_42_ levels through cross-race meta-analysis; the *KLKB1* variant replicated internally using 1,014 additional participants with exome chip. *ITPRIP*, *PLIN2*, and *TSPAN18* were associated with the midlife aβ_42_:aβ_40_ ratio via the T5 test; *TSPAN18* was significant via the cross-race meta-analysis, whereas *ITPRIP* and *PLIN2* were European American-specific. *NCOA1* and *NT5C3B* were associated with the midlife aβ_42_:aβ_40_ ratio and the fold-change in aβ_42_, respectively, via SKAT in African Americans. No associations replicated externally (N = 725).

**Conclusion:**

We discovered age-dependent genetic effects, established associations between vascular-related genes (*KLKB1*, *F12*, *PLIN2*) and midlife plasma aβ levels, and identified a plausible Alzheimer’s Disease candidate gene (*ITPRIP*) influencing cell death. Plasma aβ concentrations may have dynamic biological determinants across the lifespan; plasma aβ study designs or analyses must consider age.

## Introduction

Alzheimer’s disease (AD) is a major public health burden, afflicting 5.1 million Americans aged 65 or older; the number of cases is expected to triple by 2050, costing the nation $1.1 trillion [1]. Although there are no effective treatments to prevent, slow, or cure AD, researchers have made considerable progress in dissecting its genetic etiology and revealing biological pathways that may contain druggable targets. The International Genomics of Alzheimer’s Project leveraged results from genome-wide association studies (GWAS) of late-onset AD (LOAD) to identify candidate therapeutic targets in the immune response, endocytosis, cholesterol transport, and protein ubiquitination pathways [2]. GWAS and sequencing studies have nominated variants (representing ≈30 genes) from the whole allele frequency spectrum [3–7], yet there is a substantial proportion of LOAD heritability unexplained [6, 7]. The identification of additional therapeutic candidates is hindered by the reduced power of case-control outcomes [8].

More recent studies have employed endophenotypes of particular facets of AD pathophysiology to identify known and novel candidate genes using moderate sample sizes [9–12]. Capitalizing on the critical role that amyloid-β (aβ) plays in AD pathophysiology [12], along with functional information linking most AD-associated genes to aβ production and clearance [4], genome-wide association studies have employed cerebrospinal fluid (CSF) and plasma aβ concentrations as endophenotypes [9–12]. Plasma levels are less expensive and invasive to measure than CSF levels, are associated with brain aβ [13–16], and may capture any shared mechanisms regulating aβ processes (production, secretion, degradation, and clearance) throughout the body [12, 17]. However, their utility as AD endophenotypes is questionable. Only a handful of prospective studies and systematic meta-analyses have associated plasma aβ_42_ levels or the aβ_42_:aβ_40_ ratio with LOAD [18–23], while a paucity of associations have been documented between plasma aβ traits and AD linkage and GWAS findings [17, 24, 25]. Furthermore, plasma aβ concentrations reflect a wide array of tissue sources [26] and age-related health conditions, such as subcortical white matter lesions, cerebral microbleeds, hypertension, diabetes, infarcts, ischemic heart disease, and chronic kidney disease [14, 27, 28], a few of which are themselves associated with AD. This fosters ambiguity in the pathophysiological interpretation (AD or non-AD) of any findings.

Therefore, before exploiting plasma aβ concentrations in genetic or epidemiological studies of AD, a more comprehensive assessment of their age-dependent biological determinants is needed [27]. Deciphering the dynamic genetic architecture of plasma aβ concentrations could simultaneously implicate pathophysiological processes, pathways, and mechanisms contributing to aβ plaque accumulation in the asymptomatic preclinical and progressive phases of AD [29, 30] while suggesting novel health conditions that alter the association between plasma aβ and AD in epidemiological studies. This vital information may clarify the inconsistencies in reported associations between plasma aβ and AD across studies, as well as provide insight into the role (if any) of plasma aβ in AD pathogenesis and the utility of plasma aβ concentrations as AD biomarkers.

To systematically assess the dynamic biological determinants of plasma aβ, we performed an exome-wide association study of plasma aβ concentrations measured during two different life stages. The goal was to identify genes influencing plasma aβ_42_ concentrations and aβ_42_:aβ_40_ ratios in late middle age, old age, or change over time. We performed single-variant and gene-based association analyses using whole exome sequence from 1,414 Atherosclerosis Risk in Communities-Neurocognitive Study (ARIC-NCS) participants. This study was well-suited for this investigation because: 1) participants had two amyloid measurements spaced an average of 18 years apart, with mean ages of 59 and 77 years for the two blood draws; 2) the sample included both European (EAs) and African Americans (AAs), allowing exploration of rare population-specific variants that may contribute to health disparities; 3) the sample was enriched for dementia and mild cognitive impairment, and hence should be enriched for variants contributing to aβ accumulation; and 4) the availability of whole exome sequence permitted the analysis of both common and rare variants, enabling the first systematic interrogation of rare variants for plasma aβ levels. This investigation expanded upon the lone published GWAS meta-analysis of plasma aβ concentrations [12] which focused on cross-sectional measurements in non-demented elderly participants of European ancestry.

## Subjects and methods

### Subjects

ARIC was initiated in 1987 as a population-based cohort study of 15,792 middle-aged (45–64 years) participants drawn from four US communities (Washington County, MD; Forsyth County, NC; Jackson, MS; and suburban Minneapolis, MN) [31]. Four study visits were completed by 1999, with a fifth visit (ARIC-NCS; N = 6,538) conducted in 2011–2013 [32]. Plasma aβ, the phenotype for this investigation, was quantified on a subset (N = 2,588) of ARIC-NCS enriched for cognitive impairment. The plasma aβ sample included all individuals exhibiting impaired cognitive status (defined as low mini-mental status exam score or low standardized score on any of five cognitive domains accompanied by cognitive decline on longitudinally administered tests) during the fifth exam, all participants with a brain MRI from a prior ARIC exam, and an age-stratified (<80 years, ≥80 years) random sample of the remaining cognitively normal participants from each field center. This investigation focused on 1,414 AA and EA participants with whole exome sequence, covariates, and amyloids measured at the third (1993–1995) and fifth visits. Of these participants, 152 (72 AAs and 80 EAs) had dementia and 560 (152 AAs and 408 EAs) had mild cognitive impairment by the fifth visit; 79% of these dementia and cognitive impairment cases were ascribed to AD as the primary etiology. Cognitive diagnoses were adjudicated at the fifth visit using cognitive, neurologic, and brain imaging assessments (comprehensive diagnostic details are given in [32]). Cognitive status was not available on the whole sample at the third visit. The ARIC study has been approved by the Institutional Review Board at each field center, namely Wake Forest Baptist Medical Center (Forsyth County, NC), University of Mississippi Medical Center (Jackson, MS), University of Minnesota (suburban Minneapolis, MN), and Johns Hopkins University (Washington County, MD). Participants provided written informed consent prior to each examination.

### Plasma amyloid-β ascertainment

Amyloid quantification was performed by the Department of Molecular Pharmacology and Experimental Therapeutics at Mayo Clinic, Jacksonville, FL, from August to December 2014. The INNO-BIA assay (INNOGENETICS N.V, Ghent, Belgium) required 69 plates to measure aβ_42_ and aβ_40_ levels in both races; for each participant, the same plate was used to simultaneously measure the amyloid levels at the third and fifth visit. Beads (xMAP microspheres; conjugate 1A) bound to aβ_40_ and aβ_42_ emitted fluorescence detected by the Luminex 200 IS Total system. A five-parameter logistic regression model related the fluorescence intensities of six standards to their known amyloid concentrations. The resultant model predicted the concentrations of aβ_40_ and aβ_42_ from the measured fluorescence intensities in the samples. Intensities outside the range of the standards could not be inferred. For the single-visit analyses of aβ_42_, samples with intensities below the minimal detectable level were assigned the threshold concentrations (12 pg/ml). These individuals were omitted from the analysis of fold-changes in aβ_42_ and all analyses of the aβ_42_:aβ_40_ ratio since their ranks relative to those with measured values were inconclusive; 98 (36 AAs, 62 EAs), 17 (6 AAs, 11 EAs), 107 (41 AAs, 66 EAs), and 105 (41 AAs, 64 EAs) participants were omitted from the ratio at visit 3, the ratio at visit 5, the fold-change in the ratio, and the fold-change in aβ_42_ analyses, respectively, due to subthreshold intensities.

### Whole exome sequencing

DNA samples were assembled into Illumina paired-end pre-capture libraries; the oligonucleotide sequences and protocol are available on the Baylor College of Medicine Human Genome Sequencing Center (HGSC) website (http://www.hgsc.bcm.edu/content/protocols-sequencing-library-construction). Two, four, or six pre-capture libraries were pooled together, hybridized to the HGSC VCRome 2.1 design [33] (42Mb, NimbleGen), and sequenced in a single lane on the Illumina HiSeq 2000 or the HiSeq 2500 platform. The HGSC Mercury pipeline (https://www.hgsc.bcm.edu/content/mercury) conducted the Illumina sequence analysis while the Consensus Assessment of Sequence and Variation program de-multiplexed the pooled samples. The Burrows-Wheeler Alignment [34] algorithm mapped reads to the Genome Reference Consortium Human Build 37 (GRCh37) sequence, producing Binary Alignment/Map (BAM) files. Aligned reads were then recalibrated using the Genome Analysis ToolKit [35], BAM sorting, duplicate read marking, and realignment near insertions or deletions (indels). The Atlas2 [36] suite called both single nucleotide variants (SNVs) and indels, generating high-quality variant call files.

The ARIC exome sequence was quality controlled as part of the Cohorts for Hearts and Aging Research in Genomic Epidemiology (CHARGE) consortium. SNVs and indels were centrally filtered for posterior probabilities<0.95, variant read counts<3, variant read ratios <0.25 or >0.75, total read depths of the references<10-fold, mappability scores<0.8, missing rates>20%, mean coverage depths>500-fold, race-specific Hardy-Weinberg Equilibrium p-values<5x10^-6^, and strand bias (>99% variant reads in a single strand direction). In addition, SNVs with total coverage<10-fold and indels with total coverage<30-fold were excluded. Individuals were excluded for sex-mismatch, missingness>20%, singleton counts of 0, or values>6 standard deviations from the mean for singleton counts, race-specific mean depths, TiTv ratios, or heterozygote to homozygote ratios. The mean depth of coverage was 109.6 and 82.7 for AAs and EAs, respectively.

The annotation file included quality-controlled variants observed in at least one available exome sequencing project (e.g. CHARGE, the NHLBI Exome Sequencing Project). Variants were annotated using ANNOVAR [37] and dbNSFP v2.0 according to GRCh37 and RefSeq. This multiple study SNPinfo file was used as a component of the R package seqMeta (http://cran.rproject.org/web/packages/seqMeta/index.html).

### Covariates

We included age, sex, and apolipoprotein-E (*APOE)* as covariates in this investigation. *APOE* genotypes were determined using TaqMan assays and the ABI 7700 Sequence Detection System (Applied Biosystems, Foster City, CA). *APOE ε*4 carrier status (0 = No, 1 = Yes) indicated whether an individual carried at least one copy of the ε4 allele; this carrier status was strongly associated (minimum p-value of 1x10^-8^ for aβ_42_ at visit 5) with age- and gender-adjusted plasma aβ levels in EAs and was moderately (p-values in the range of 0.1 to 0.2) associated in AAs. Although the sample included *APOE* ε4 homozygotes (23 (6%) and 20 (2%) in the AAs and EAs, respectively), *APOE ε*4 carrier status tended to fit better (by Akaike Information Criterion in models of age- and gender-adjusted plasma aβ) than separate coefficients for ε4 heterozygotes and homozygotes or the number of ε4 alleles.

### Population structure

Race-specific principal components (PCs), the first ten of which were utilized to control for population stratification in the statistical analysis, were calculated from the genotype data using Eigenstrat [38]. Variants with minor allele frequencies (MAFs)<0.05, missing rates>0.05, or Hardy-Weinberg Equilibrium p-values<1x10^-5^ were excluded before pruning the variants for linkage disequilibrium (r^2^) >0.3. In total, 29,551 and 16,323 SNVs were used to construct the PCs in AAs and EAs, respectively.

### Statistical methods

Single-visit amyloid measures were linearly regressed onto age, gender, and *APOE ε*4 carrier status separately by race (field center was not statistically significant). The fold-change amyloid traits were regressed onto the age at the third visit, the time elapsed between visits, gender, and *APOE* ε4 carrier status. Following rank-based inverse normal transformation of the residuals, we used seqMeta (version 1.5; http://cran.r-project.org/web/packages/seqMeta/) to conduct race-specific single-variant and gene-based association tests while adjusting for population structure (the first ten PCs). Linear regression models, assuming additive genetic effects, were fit on all autosomal SNVs with MAF≥0.01. T5 burden and Sequence Kernel Association (SKAT; using default “Wu” weights) gene-based tests were conducted on functional (nonsynonymous, splicing, stop gain, stop loss, or frameshift) autosomal variants with MAF ≤ 0.05. For both the single-variant and gene-based tests, we meta-analyzed the African and European American results (score statistics and genotype covariance matrices) using fixed effects models in seqMeta. We applied a Bonferroni correction for the number of unique variants (up to 113,423) or genes (up to 16,733) tested per trait (S1 Table contains the number of variants/genes tested per trait), yielding exome-wide significance thresholds of 4.41x10^-7^ (0.05/113,423) and 2.99 x10^-6^ (0.05/16,733) for the single-variant and gene-based tests, respectively. The quantile-quantile plots from the race-specific analyses and the cross-race meta-analyses exhibited minimal inflation and were well-behaved for both the single-variant (genomic inflation factors ranged from 0.99 to 1.03; S1 Fig) and gene-based tests (both overall and by minor allele count (MAC) thresholds; S2–S7 Figs).

### Replication

We internally replicated significant single-variant tests using 1,014 ARIC participants with exome chip (Illumina Human Exome BeadChip v1.0) but not exome sequence data; these individuals had plasma aβ measured at both the third and fifth visits and were used to replicate both cross-sectional and change over time traits. The genotype calling and quality-control procedures have been described elsewhere [39], while the statistical methods matched those of the sequence analyses. We did not internally replicate significant gene-based tests because few functional SNVs (≤ 2) were available in the genes of interest using exome chip participants from the appropriate racial group. We externally replicated both the significant single-variant and gene-based tests using 725 participants from the Framingham Heart Study (FHS) Offspring and Third Generation (Gen 2 and Gen 3) cohorts [40, 41]; these participants had amyloids measured once and were used to replicate cross-sectional findings only. All significant cross-sectional associations from the discovery phase were with midlife plasma aβ levels (i.e. from the third visit in ARIC), thus we restricted the FHS replication sample to participants aged 50–70 years (mean age of 60±6 years, 48% female). The amyloid assessment was consistent between FHS and ARIC (the INNO-BIA assay conducted at the same lab), as was the exome sequence calling, quality control, and annotation (both members of CHARGE). The single-variant and gene-based analyses of the inverse-transformed amyloids incorporated a kinship matrix to account for FHS family structure and included significant PCs.

## Results

The discovery analysis included 406 AAs and 1,008 EAs with whole exome sequence (Table 1; for summary statistics by cognitive status, see S2 and S3 Tables). Only one-third of AAs were male, whereas about half (47%) of EAs were male. Both races had similar age distributions, with the third visit corresponding to late middle age and the fifth visit corresponding to older age, and high *APOE* ε4 carriage rates compared to the general population (20–25%) [42]. In both AAs and EAs, mean aβ_42_ levels increased between visits and mean aβ_42_:aβ_40_ ratios decreased between visits. Although the third visit aβ_42_ levels differed between the races, the mean fold-changes were similar. The internal (ARIC exome chip) and external (FHS exome sequence) replication samples had lower *APOE ε*4 carriage rates (34% for ARIC AAs, 18% for ARIC EAs, and 22% for FHS EAs) than the discovery samples (S4 Table**)**. The FHS replication sample included only EAs and had more males (52%) and higher midlife plasma aβ levels (both aβ_42_ and aβ_42_:aβ_40_ ratio) than all ARIC samples.

**Table 1:** Descriptive statistics of sequenced ARIC participants.

Characteristic	African Americans (N = 406)	European Americans (N = 1,008)
% Female	67.7%	53.4%
% *APOE* ε4 carriers	44.1%	33.0%
Time between amyloid measurements (in years)		
Mean (SD)	18 (1)	18 (1)
Range	16–20	16–20
Age (in years)		
Visit 3 Mean (SD)	59 (5)	60 (5)
Range	50–71	50–70
Visit 5 Mean (SD)	77 (5)	77 (5)
Range	67–89	67–90
Plasma aβ_42_ (in pg/ml)		
Visit 3 Mean (SD)	26.69 (9.30)	30.06 (9.64)
Interquartile Interval	20.10–31.80	23.20–36.34
Visit 5 Mean (SD)	33.57 (12.06)	38.45 (11.23)
Interquartile Interval	25.50–39.70	30.70–45.20
Fold-change Mean (SD)	1.30 (0.49)	1.31 (0.41)
Interquartile Interval	1.04–1.44	1.06–1.47
aβ_42_:aβ_40_ ratio		
Visit 3 Mean (SD)	0.20 (0.11)	0.19 (0.07)
Interquartile Interval	0.15–0.22	0.15–0.23
Visit 5 Mean (SD)	0.17 (0.07)	0.17 (0.06)
Interquartile Interval	0.13–0.20	0.13–0.19
Fold-change Mean (SD)	0.95 (0.33)	0.93 (0.31)
Interquartile Interval	0.75–1.11	0.73–1.08

NOTE: SD = Standard deviation. The amyloid measures were non-normal, thus the interquartile interval conveyed the amyloid values in the 25^th^ to 75^th^ percentile.

### Single-variant analysis

#### ARIC exome sequence analyses

**Fig 1:**
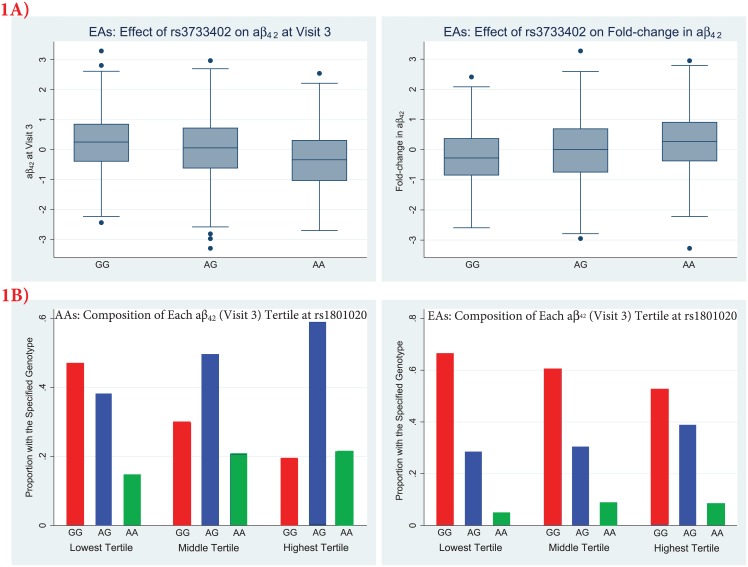
*KLKB1* and *F12* single-variant associations in ARIC participants with exome sequence. Panel A shows boxplots of the third visit aβ_42_ levels and the fold-changes in aβ_42_ stratified by the rs3733402 genotype in EAs. Panel B depicts the relative frequencies of the rs1801020 genotypes within each aβ_42_ (visit 3) tertile in AAs and EAs. The plotted values are inverse normal transformed amyloid values adjusted for age, gender, time between visits (fold-change aβ_42_ only), and *APOE* ε4 carriage status.

Kallikrein B, plasma (Fletcher factor) 1 [*KLKB1*] and coagulation factor XII (Hageman factor) [*F12*] contained common SNVs significantly (p≤4.41x10^-7^) associated with aβ_42_ traits (Table 2). Nonsynonymous SNV rs3733402 in *KLKB1* was associated with aβ_42_ levels at the third visit and the fold-change in aβ_42_ across visits; these associations were significant using the EAs alone but had the same directions of effects and moderate statistical support (p<0.09) in AAs. EAs with the A allele had lower aβ_42_ levels at the third visit but an increased fold-change in aβ_42_ over visits (Fig 1A); individuals with this allele may start late-midlife with lower levels but have larger increases over the next 16–20 years. A second SNV in *KLKB1* (rs925453) was associated with the third visit aβ_42_ levels but became insignificant after conditioning on rs3733402 (p-values of 0.27 and 0.43 in AAs and EAs, respectively), undermining its claim as an independent finding.

**Table 2:** Significant single-variant results.

Trait	Gene	SNV Rs Number (Functional Region)	Coded Allele	Chr	Position in base pairs (Build 37)	Analysis	Race	N	CAF	β	se(β)	p-value
aβ_42_ at visit 3	*KLKB1*	rs3733402 (Nonsynonymous)	A	4	187,158,034	ARIC Exome Sequence	AA	406	0.73	-0.18	0.08	0.03
							EA	1,007	0.51	-0.26	0.04	**3.55E-09**
							Meta	1,413	0.57	-0.24	0.04	**4.33E-10**
						ARIC Exome Chip	AA	149	0.69	-0.12	0.14	0.40
							EA	865	0.51	-0.19	0.05	4.02E-05
							Meta	1,014	0.54	-0.19	0.04	3.07E-05
						Meta-ARIC	ALL	2,427	0.56	-0.22	0.03	**9.90E-14**
						FHS	EA	725	0.53	0.04	0.05	0.41
	*F12*	rs1801020 (UTR5)	G	5	176,836,532	ARIC Exome Sequence	AA	406	0.57	-0.28	0.07	7.71E-05
							EA	1,007	0.76	-0.20	0.05	8.37E-05
							Meta	1,413	0.71	-0.23	0.04	**3.89E-08**
						FHS	EA	725	0.75	-0.05	0.06	0.39
Fold-change in aβ_42_	*KLKB1*	rs3733402 (Nonsynonymous)	A	4	187,158,034	ARIC Exome Sequence	AA	364	0.71	0.14	0.08	0.09
							EA	941	0.50	0.27	0.05	**9.28E-09**
							Meta	1,305	0.56	0.24	0.04	**5.03E-09**
						ARIC Exome Chip	AA	132	0.69	0.19	0.15	0.20
							EA	818	0.50	0.16	0.05	8.88E-04
							Meta	950	0.53	0.16	0.05	3.80E-04
						Meta- ARIC	ALL	2,255	0.55	0.20	0.03	**1.58E-11**

NOTE: Chr = Chromosome; N = Sample size; CAF = Coded allele frequency; β = Effect of each copy of the coded allele on the trait; se(β) = Standard error of the effect of each copy of the coded allele on the trait. SNV rs1801020 was not available on the exome chip in ARIC and was poorly imputed in the 1000 Genomes GWAS data in ARIC (IMPUTE2 imputation qualities of 0.707 and 0.606 in AAs and EAs, respectively).

Rs1801020 in the 5’ untranslated region of *F12* was significantly associated with the third visit aβ_42_ levels through cross-race meta-analysis (Table 2). Both races provided evidence of association (p<0.0001), with lower aβ_42_ levels for the G allele. The lowest tertile of aβ_42_ had the largest proportion of participants with the GG genotype in both races (Fig 1B**)**. Eight suggestive associations (4.41x10^-7^<p ≤8.82x10^-6^) with aβ were identified (S5 Table), including SNVs in *PPP5C* (rs917948), *FUT9* (rs9499636), *ECHS1* (rs2230260), *SMAP1* (rs576516), *PDZD8* (rs35664484), *RRP12* (rs6584122), *CACNA2D4* (rs202022529*)* and *ADGRF5* (rs678312).

#### Replication analyses

We internally validated the significant *KLKB1* (rs3733402) associations using 1,014 ARIC participants who had exome chip but not exome sequence data. The directions of effects on the third visit aβ_42_ levels and the fold-change in aβ_42_ levels were consistent between participants with exome chip and sequence (Table 2). The rs3733402 associations replicated in EAs alone, with strengthened evidence in the cross-race meta-analysis. We could not internally replicate rs1801020 in *F12*; it was not available on the exome chip and was poorly imputed in the 1000 Genomes GWAS data (AAs and EAs had imputation qualities of 0.707 and 0.606, respectively). Neither the *KLKB1* or *F12* variants externally replicated in FHS.

### Gene-based tests

#### ARIC exome sequence analyses

**Fig 2:**
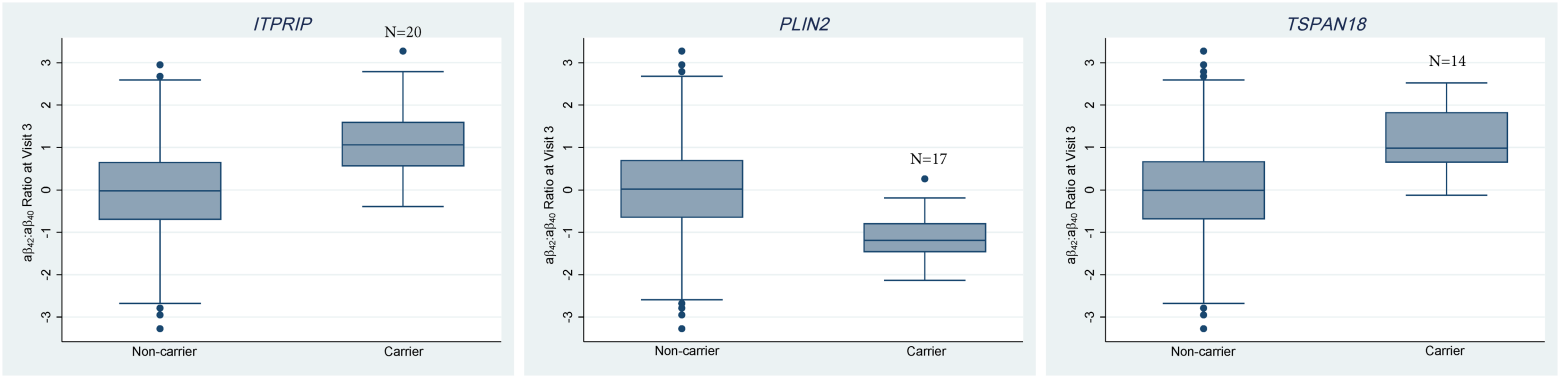
Effects of carrying a minor allele in *TSPAN18*, *ITPRIP*, and *PLIN2* on the third visit aβ_42_: aβ_40_ ratio in EAs. The plotted values are the inverse normal transformed ratios adjusted for age, gender, and *APOE ε*4 carriage status.

**Fig 3:**
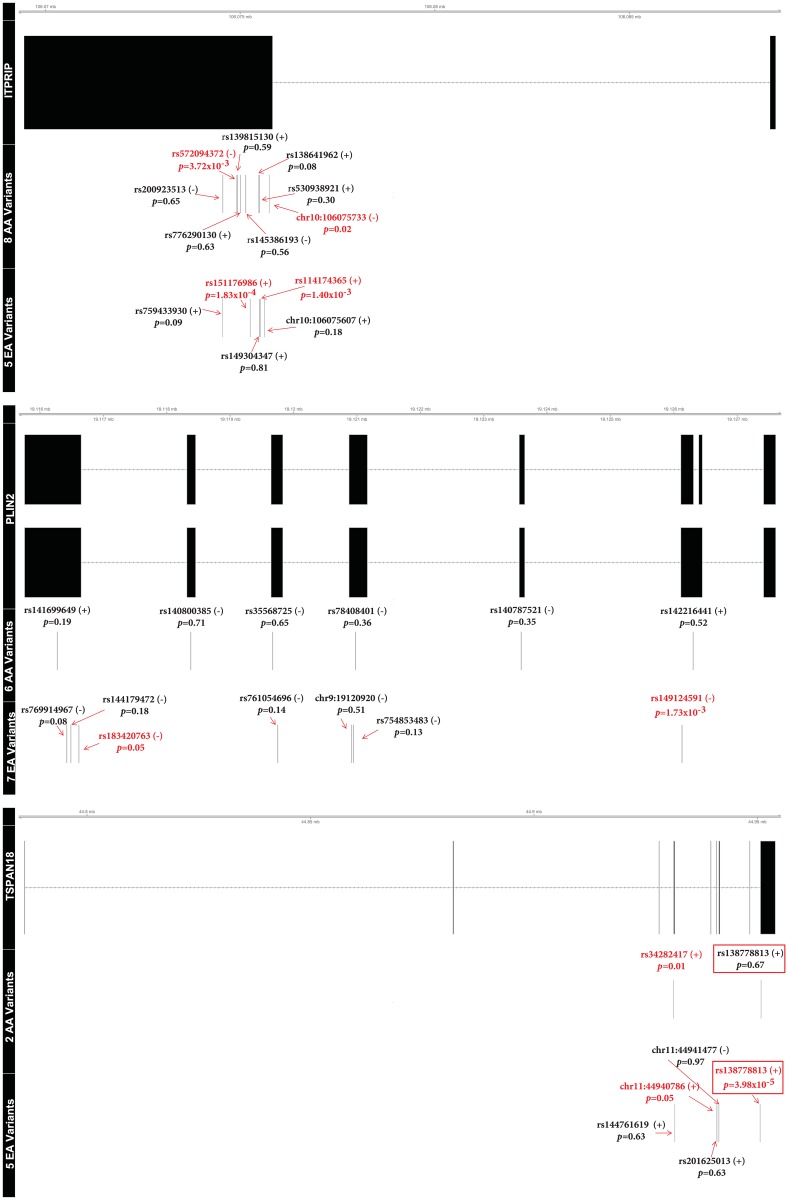
Variants contributing to the significant T5 gene-based tests. This figure depicts the single-variant model results for variants contributing to the T5 tests of *ITPRIP*, *PLIN2*, and *TSPAN18* on the third visit aβ_42_: aβ_40_ ratio. One isoform was drawn unless the regions harboring variants differed substantially. Each contributing variant is labeled by name, single-variant association p-value, and direction of the minor allele effect (in parentheses). Variants with nominal (p<0.05) levels of association are in red. Overlapping variants in the two racial groups are encapsulated by a red box.

Inositol 1,4,5-triphosphate receptor interacting protein [*ITPRIP*], perilipin-2 [*PLIN2*], tetraspanin 18 [*TSPAN18*], nuclear receptor coactivator 1 [*NCOA1*], and 5’-nucleotidase, cytosolic IIIB [*NT5C3B*] were associated (p≤2.99x10^-6^) with plasma aβ via gene-based tests (Table 3) and had cumulative MAFs ranging from 0.76% to 1.49% in the significant race-specific- or meta-analysis. *ITPRIP*, *PLIN2*, and *TSPAN18* were significantly associated with the third visit aβ_42_:aβ_40_ ratio via the T5 burden test; the *ITPRIP* and *PLIN2* associations were EA-specific with no shared variants across races, whereas *TSPAN18* showed nominal association (p<0.05) in both races, had one variant (rs138778813) shared across races, and was significant in the cross-race meta-analysis. Carrying a minor allele in *ITPRIP* or *TSPAN18* increased the third visit aβ_42_:aβ_40_ ratio in EAs whereas carrying a minor allele in *PLIN2* decreased the ratio (Fig 2). These patterns were reflected in the single-variant model coefficients of the contributing variants (Fig 3 and S6–S8 Tables); all *PLIN2* variants had negative coefficients in EAs and all but one of the *TSPAN18* and *ITPRIP* variants had positive coefficients. The lone overlapping *TSPAN18* variant (rs138778813) increased the ratio in both races but showed greater evidence of association in EAs than AAs (single-variant p-values of 3.98x10^-5^ and 0.67, respectively), possibly due to differences in the number of copies of the minor allele in the two groups (10 and 1 minor allele copies, respectively).

**Table 3:** Significant gene-based (T5 and SKAT) results.

Significant Test in ARIC	Trait	Gene	Chr	Analysis	Race	N	MAC	# SNVs	T5 Test			SKAT
									β	se(β)	p-value	p-value
T5	aβ_42_:aβ_40_ ratio at visit 3	*ITPRIP*	10	ARIC	AA	370	47	8	-0.11	0.15	0.48	0.15
					EA	945	20	5	1.13	0.23	**5.40E-07**	9.68E-05
					Meta	1,315	67	13	0.28	0.13	0.03	3.57E-03
				FHS	EA	725	10	7	0.10	0.32	0.75	0.03
		*PLIN2*	9	ARIC	AA	370	18	6	-0.14	0.25	0.57	0.65
					EA	945	17	7	-1.15	0.25	**2.94E-06**	1.02E-03
					Meta	1,315	146^a^	13	-0.33	0.08	8.59E-05	1.05E-03
				FHS	EA	725	18	6	-0.28	0.24	0.24	0.44
		*TSPAN18*	11	ARIC	AA	370	6	2	1.00	0.42	0.02	0.02
					EA	945	14	5	1.15	0.27	2.04E-05	4.29E-05
					Meta	1,315	20	6	1.11	0.23	**1.08E-06**	1.74E-05
				FHS	EA	725	25	5	-0.14	0.21	0.51	0.27
SKAT	aβ_42_:aβ_40_ ratio at visit 3	*NCOA1*	2	ARIC	AA	370	11	5	0.90	0.31	4.02E-03	**1.34E-06**
					EA	945	85	15	-0.11	0.11	0.31	0.08
					Meta	1,315	96	19	0.00	0.10	0.99	0.65
				FHS	EA	725	58	10	-0.06	0.13	0.64	0.46
	Fold-change in aβ_42_^b^	*NT5C3B*	17	ARIC	AA	364	7	4	1.35	0.38	4.40E-04	**5.67E-07**
					EA	941	9	5	0.02	0.31	0.94	0.88
					Meta	1,305	16	9	0.54	0.24	0.02	3.18E-06

NOTE: Chr = Chromosome; N = Sample size; MAC = Number of copies of the minor alleles; # SNVs = Number of SNVs contributing to the gene-based test; β = Effect of the minor alleles on the trait; se(β) = Standard error of the effect of the minor alleles.

^a^One of the *PLIN2* SNVs (rs35568725) had a minor allele frequency of 0.059 in EAs but 0.005 in AAs, thus was included in the meta-analysis (combined minor allele frequency 0.044) for both cohorts by default; we performed the meta-analysis both including and omitting this variant in the EA group. Including this variant in both racial groups yielded a MAC of 146, a T5 meta-analysis p-value of 8.59E-05 (β = -0.33, se(β) = 0.08), and a SKAT meta-analysis p-value of 1.05E-03. Excluding this variant in the EAs yielded a minor allele count of 35, a T5 meta-analysis p-value of 2.12E-04 (β = -0.64, se(β) = 0.17), and a SKAT meta-analysis p-value of 3.36E-03.

^b^ Fold-change in aβ_42_ was not available in FHS.

**Fig 4:**
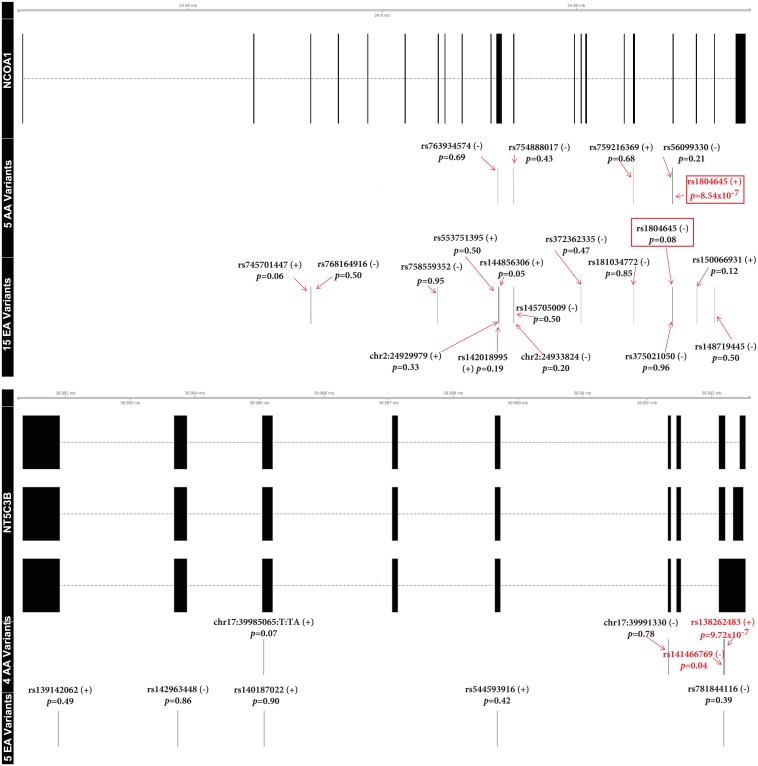
Variants contributing to the significant SKAT gene-based tests. This figure displays the single-variant model results for variants contributing to the SKAT tests of *NCOA1* and *NT5C3B* on the third visit aβ_42_: aβ_40_ ratio and fold-change in aβ_42,_ respectively. One isoform was drawn unless the regions harboring variants differed substantially. Each contributing variant is labeled by name, single-variant association p-value, and direction of the minor allele effect. Variants with nominal (p<0.05) levels of association are in red. Overlapping variants in the two racial groups are encapsulated by a red box.

*NCOA1* and *NT5C3B* were associated with the third visit aβ_42_:aβ_40_ ratio and the fold-change in aβ_42_ levels, respectively, by SKAT (Table 3). These associations were AA-specific and had one major contributing SNV each. Rs1804645 (*NCOA1*; p = 8.54x10^-7^; 6 minor allele copies) and rs138262483 (*NT5C3B*; p = 9.72x10^-7^; 4 minor allele copies) were the primary contributors (Fig 4 and S9 and S10 Tables). No *NT5C3B* variants overlapped races but *NCOA1* had one variant (rs1804645) with opposite directions of effects and different allele frequencies in AAs and EAs (MAF of 0.008 and 0.037, respectively). Sixteen additional genes (*ABL2*, *ATP2A1*, *CCDC102B*, *CSHL1*, *DSG4*, *FGF23*, *FLNB*, *GALNT13*, *LMF2*, *MED25*, *NIPAL2*, *NUDT7*, *SNAPC1*, *TARSL2*, *TMEM202*, *TRUB2*) were suggestively (2.99 x10^-6^ < p ≤ 5.98x10^-5^) associated with amyloids through the T5 or SKAT tests (S11 and S12 Tables).

#### Replication analyses

Of the four genes associated with the third visit aβ_42_:aβ_40_ ratio, none strictly replicated in FHS cohorts (Table 3). However, *ITPRIP* was nominally (p-value≤0.05) associated using SKAT instead of the T5 burden test. Rs114174365, the only *ITPRIP* variant with more than one minor allele copy in both ARIC and FHS EAs, had similar effect sizes (0.89 and 1.01) in both, with p-values 0.001 and 0.045, respectively (S13 Table). *PLIN2*, *NCOA1*, and *TSPAN18* did not even nominally replicate. The largest contributing *TSPAN18* variant (rs138778813; 10 and 21 minor allele copies in ARIC and FHS EAs) had opposite directions of effects and different allele frequencies (0.005 and 0.015, respectively) in the two cohorts, possibly due to study design issues (relateds in FHS, required survival to older age and oversampling for cognitive impairment in ARIC). We could not replicate the association between *NT5C3B* and the fold-change in aβ_42_ because FHS had only one amyloid assessment available.

### Temporal sensitivity of associations

We compared the exome-wide p-values from the third and fifth visits using scatterplots but found no systemic patterns (such as the third visit yielding lower p-values than the fifth visit, etc.) for either aβ_42_ or the ratio using single-variant or gene-based tests. The ranks of the p-values at the two time points were only weakly correlated (Spearman correlation coefficients ranging from 0.14 to 0.34). Our significant and suggestive findings demonstrated temporal sensitivity; no findings were significant or suggestive at both visits (S14–S18 Tables). Only half (*F12*, *PLIN2*, *NCOA1*) of the significant third visit associations showed nominal evidence at the fifth visit. The effect of rs3733402 (*KLKB1*) on aβ_42_ levels was near zero (p-values>0.620) for all fifth visit analyses. Rs1801020 (*F12*) was nominally associated with aβ_42_ in the fifth visit EA- and meta-analyses (p-values 0.014 and 0.036, respectively) but the effect estimates were attenuated (-0.126 and -0.088, respectively) compared to the third visit (-0.201 and -0.229, respectively). Similar results were observed for the significant gene-based tests.

### Phenotype-specificity of associations

All significant associations were specific to one type of amyloid measure (aβ_42_ or the ratio; see S19 and S20 Tables) and failed to produce even suggestive evidence for the other. For the two common variants associated with aβ_42_ traits (rs3733402 in *KLKB1* and rs1801020 in *F12*), the fold-change was the ratio measure with the strongest cross-race evidence (meta-analysis p-values of 0.005 and 1.46x10^-4^ and consistent direction of effects across AAs and EAs). We also note that two of the ratio-associated genes (*ITPRIP* and *NCOA1*) failed to produce nominal associations (p-value<0.05) for any aβ_42_ trait in any analysis (AA-specific, EA-specific, or meta-analysis).

## Discussion

Leveraging late-midlife plasma aβ concentrations and fold-changes from a moderate-sized biracial sample enabled the identification of seven genes that were obscured in cross-sectional analyses of the elderly. These findings echoed those of the published GWAS meta-analysis of plasma aβ concentrations which failed to identify significant genetic variants in non-demented elderly participants [12]. Plasma aβ concentrations change with age, at least until dementia or brain plaques appear [22, 43], and could have a dynamic genetic architecture across the lifespan. Such age-dependent genetic associations have been reported for other complex traits, including blood pressure [44, 45] and lipids [46], and can represent genes that are active at specific ages or genes that are active across the age spectrum with varying effect magnitudes [45]. Accounting for age-dependent genetic effects, whether through model-based approaches or age stratification, can enhance gene discovery efforts and our understanding of intraindividual variation in plasma aβ levels [44–46]. Such analyses may suggest the optimal age range to capture particular facets of disease (e.g. AD, vascular) pathophysiology while negating the need for massive sample sizes.

Numerous phenomena could reshape the genetic landscape with aging. Gene expression and posttranslational protein modification are dynamic processes that change with age [45]. An accumulation of behavioral and environmental exposures can cause epigenetic changes, such as DNA methylation, histone modification, and microRNA expression, thereby altering gene expression (and hence the detectable genetic effects present) across the lifespan [47]. Age-related oxidative damage may mediate an accumulation of posttranslational modifications to proteins [48], altering their function and the effects of genes encoding them. More specific to our phenotypes, age-related health conditions may increase the heterogeneity of genetic effects contributing to plasma aβ levels, thereby changing the genetic underpinnings of these traits with age; subcortical white matter lesions, cerebral microbleeds, hypertension, diabetes, infarcts, ischemic heart disease, and chronic kidney disease have been associated with plasma aβ_42_ levels or the ratio [14, 27, 28]. Gene-age interactions could arise from differences in the aβ processes perturbed (production, degradation, transportation across the blood brain barrier, reabsorption through the CSF, or clearance from the body [49–53]) or tissue sources (platelets, skeletal muscle, pancreas, kidney, liver, vascular wall, lung, intestine, skin, glands, and brain [26]) across the lifespan. For example, the genetic underpinnings of plasma aβ could mimic the pattern hypothesized for early- versus late-onset AD [26]; genes involved in aβ production may have a greater impact on plasma concentrations in the young, while genes involved in aβ clearance may have a greater impact in the elderly.

Three gene-based associations (*ITPRIP*, *TSPAN18*, and *PLIN2*) had decent minor allele counts in the discovery sample and plausible connections to plasma aβ. The protein encoded by *ITPRIP* binds to the inositol-1,4,5-triphosphate receptor (IP_3_R) and allosterically downregulates IP_3_-induced calcium release from the endoplasmic reticulum [54]. Oligomeric aβ_42_ alters IP_3_-triggered calcium release [55] and, in return, IP_3_-induced calcium release may influence amyloid precursor protein (APP) cleavage to aβ_40_ and aβ_42_ [56]. Thus, ITPRIP may interact with IP_3_R to modulate both the aβ concentration and synaptic plasticity through calcium signaling [54]. A pathway analysis of plasma aβ GWAS results lent credibility to our *ITPRIP* finding [12]; eighteen of twenty-seven Ingenuity canonical pathways associated with plasma aβ contained the receptor (IP_3_R) modulated by ITPRIP. Located near a previously reported AD linkage peak [57], *ITPRIP* is an attractive AD candidate therapeutic target because of its role in cell death; it binds and inhibits the activity of death-associated protein kinase, a key component in cell death signaling pathways [58]. *Itprip* knock-out mice show increased cell death [58]; the effect on neuronal death is quite pronounced, with *Itprip* knock-out hippocampal neurons exhibiting 28% survival of wild-types after exposure to stimuli [58]. Reduction of IP_3_R–mediated calcium signaling rescued presenilin-associated AD pathogenesis in mouse models [59], thus drugs that enhance ITPRIP activity may prevent cell death in neurodegenerative diseases and stroke [58].

*TSPAN18*, supported by both races in ARIC, encodes a four-transmembrane protein from a highly conserved family known to influence the spatial organization of membrane proteins through interactions with each other, signaling proteins, enzymes, transmembrane receptors, and adhesion molecules [60]. Tetraspanins also participate in cellular trafficking through endocytic and recycling organelles, lysosomes, or secreted vesicles [61, 62]. Up- or down-regulation of tetraspanin proteins and microdomains influences aβ production, specifically through α- and γ-secretase processing of APP [63]. The tetraspanin associated with plasma aβ in this investigation, *TSPAN18*, has been inconsistently associated with schizophrenia in Han Chinese [64, 65], while other tetraspanins have been associated with schizophrenia, bipolar disorder, and X-linked mental retardation [66, 67].

*PLIN2* encodes perilipin-2, the most prevalent lipid-droplet-associated protein in nonadipose tissue [68]. Perilipin-2 is associated with lipid droplet biogenesis, cholesterol efflux, plasma very low-density lipoprotein cholesterol levels, and intracellular and plasma triglyceride levels [68–70]. The association between aβ and *PLIN2* is plausible since cholesterol concentrations and trafficking impact APP processing and aβ degradation [71, 72] and lipid droplet presence is positively correlated with aβ levels in brain neurons from AD patients [73]. The association between *PLIN2* and plasma aβ may be due to atherosclerosis [70, 74, 75]; perilipin-2 aids foam cell formation [74] and is overexpressed in atherosclerotic plaques [70] which produce aβ [76]. The overexpression of *PLIN2* in macrophages increases the expression of monocyte chemoattractant protein-1 [77], which itself elevates aβ accumulation in AD mice [78]. The association between *PLIN2* and plasma aβ may also be due to a host of metabolic disorders, such as fatty liver disease and obesity, which are associated with perilipin-2 and aβ levels [79, 80]. We note that *PLIN2* is near a well-known AD linkage peak on chromosome 9p22.1 [81].

Single-variant tests linked two key members (*F12* and *KLKB1)* of the kallikrein-kinin system to plasma aβ. *F12* and *KLKB1* encode proteins that participate in complement activation, blood clotting, neutrophil aggregation, fibrinolysis (through plasminogen activation), and the bioprocessing of vasoactive peptides [82]. Most notably, contact activation of coagulation factor XII (FXII; encoded by *F12*) causes cleavage of plasma prekallikrein (encoded by *KLKB1*) to kallikrein, liberating bradykinin from high molecular weight kininogen [83]. The significant variants in *F12* (rs1801020) and *KLKB1* (rs3733402) have documented biological consequences. The A allele at Rs1801020, which is four bases upstream of the translation initiation codon, decreases translation efficiency and plasma FXII activity [84]. The G allele at rs3733402 substitutes serine for asparagine in an apple domain that mediates the binding of plasma prekallikrein to high molecular weight kininogen [83]. Thus, rs3733402 and rs1801020 are expected to alter bradykinin levels; they have also been associated with levels of plasma renin [85], biological surrogates of endothelin-1 and adrenomedullin [86], and B-type natriuretic peptide (rs3733402 only)[87]. The renin-angiotensin and endothelin-1 signaling pathways have been associated with plasma aβ via a pathway analysis of GWAS results [12], somewhat corroborating our common variant associations.

There are several potential mechanisms connecting the kallikrein-kinin system to aβ. Bradykinin, endothelin-1, and angiotensin-1 (a product of the renin cascade) are substrates for the aβ-degrading proteases neprilysin, endothelin-converting enzyme, and angiotensin converting enzyme, respectively [53], possibly influencing aβ degradation rates. Or, perhaps, bradykinin could participate in a feedback loop to regulate aβ_42_ levels; aβ_42_ may interact with FXII to increase bradykinin levels while bradykinin may decrease the formation of aβ_42_ through α-secretase processing of APP [88–91]. Whether the association between plasma aβ and the kallikrein-kinin system reflects any AD pathophysiology is unknown [92]. Variants in *KLKB1* or *F12* were not identified in the largest GWAS of AD to date [3], although the kallikrein-kinin system is overactivated in the plasma, CSF, and frontal and temporal cortices of AD cases [90, 92, 93] and is most prominently expressed in brain regions with the earliest signs of AD [94]. FXII, its binding sites, and components of its proteolytic cascades are present in aβ plaques of autopsied brains [95]. In addition, studies have shown that bradykinin can incite τau protein phosphorylation and subsequent learning and memory impairments [96], induce IP_3_ accumulation and mobilization of intracellular calcium [97], and impact inducible nitric oxide synthase, resulting in cognitive impairment [94].

We surmise that *KLKB1* and *F12* could be associated with both vascular disease and aβ deposition, with subsequent effects on AD. Pairwise associations between kallikrein-kinin system genes (*KLKB1* and *F12)*, complex vascular traits (hypertension, myocardial infarction, and stroke), and plasma aβ (aβ_42_ or the ratio) concentrations have been reported [14, 27, 98–101]. In turn, midlife vascular risk factors have been linked to both cognitive decline and brain amyloid deposition in late-life [102, 103], while the latter has been associated with plasma aβ concentrations [13–16]. The association of vascular-related genes with midlife, but not late-life, plasma aβ and the association of midlife, but not late-life, vascular risk factors with late-life brain amyloid deposition [103], bolsters the assertion that genetic studies of midlife plasma aβ levels can capture contributors to late-life amyloid deposition.

Although several of the observed plasma aβ genetic associations were supported by in vitro studies and animal models, external replication is required. This is particularly true for the tenuous AA-specific findings in *NT5C3B* and *NCOA1* which had smaller minor allele counts (S21 Table details the functions of these genes and their possible connections to aβ). Unfortunately, our replication efforts were hindered by the paucity of studies with plasma aβ and exome sequence in a large number of middle-aged (especially underrepresented minority) participants. Relying on a single sample of 725 EAs exacerbated the already difficult task of replicating single-variant and gene-based tests [6]. Few functional variants overlapped across ARIC and FHS (≤3 per gene within race, ≤1 per gene across races) and the study designs differed. ARIC included AAs and EAs who survived until the fifth examination and were oversampled for cognitive impairment. In contrast, FHS included EAs who attended the visit of interest, without enriching for future cognitive status. Thus, non-replication could indicate false positives due to the complicated ARIC sample selection strategy and attrition. As is common practice in agnostic gene identification studies, we ignored these issues in the analyses which biased the estimated effects but allowed us to find and prioritize candidate genes for further study.

Our investigation had a few limitations. The blood samples from the third visit were stored for two decades before amyloid assessment whereas the fifth visit samples were only stored short term. Therefore the genetic associations observed in the third visit and the change over time may not be due to the age effects but rather genes influencing plasma aβ levels (via degradation) over long term storage. This concern is somewhat minimized by the fact that aβ levels are stable for at least one year and three freeze-thaw cycles [104] and INNO-BIA assays have been used on samples stored for one to two decades [19, 105]. Some participants, potentially containing rare aβ-associated variants with large effects, had amyloid concentrations below the minimal detection threshold. The exclusion of these participants from the ratio and fold-change analyses may have precluded the identification of additional aβ-associated genes. Our primary analysis ignored the amyloid assay plate effects, included low-frequency (0.01<MAF≤0.05) variants in the burden test, and ignored interactions between variants/genes and *APOE* ε4 carriage or future cognitive status. Sensitivity analyses showed that these minimally impacted the significant findings (see S22–S26 Tables for analyses incorporating batch effects, restricting to rare variants (MAF≤0.01), and stratifying by *APOE ε*4 and cognitive impairment), although the magnitude of the single-variant effects tended to be larger among those who became cognitively impaired. Lastly, we used residuals of fold changes as a simplistic outcome in this investigation but will conduct future studies using more sophisticated longitudinal analyses.

Overall, this investigation highlighted the potential age-dependency of plasma aβ genetic associations, established connections between midlife plasma aβ levels and vascular-associated genes, and suggested novel candidate AD genes for further study. Our findings implicate complex traits (e.g. hypertension) that are associated with both plasma aβ and AD. Therefore, we must be cognizant that differences in non-AD complex trait distributions may confound the association between plasma aβ and AD across studies. A more comprehensive understanding of the biological contributors to plasma aβ across the lifespan is critical to understand their role (if any) in AD pathogenesis and their utility as AD biomarkers.

## Supporting information

S1 Fig QQ plots for single-variant tests using ARIC participants with exome sequence. (PDF)

S2 Fig QQ plots for T5 burden tests using ARIC participants with exome sequence. (PDF)

S3 Fig QQ plots for SKAT using ARIC participants with exome sequence. (PDF)

S4 Fig QQ plots for the T5 test of the third visit aβ_42_:aβ_40_ ratio in EAs using different minor allele count thresholds (starting at 0.5% cumulative MAF). (PDF)

S5 Fig QQ plots for the T5 test of the third visit aβ_42_:aβ_40_ ratio from the cross-race meta-analysis using different minor allele count thresholds (starting at 0.5% cumulative MAF). (PDF)

S6 Fig QQ plots for the SKAT test of the third visit aβ_42_:aβ_40_ ratio in AAs using different minor allele count thresholds (starting at 0.5% cumulative MAF). (PDF)

S7 Fig QQ plots for the SKAT test of the fold-change in aβ_42_ in AAs using different minor allele count thresholds (starting at 0.5% cumulative MAF). (PDF)

S1 Table Number of variants or genes tested for each trait in the discovery analysis. (XLSX)

S2 Table Mean plasma aβ levels by cognitive status in African Americans. (XLSX)

S3 Table Mean plasma aβ levels by cognitive status in European Americans. (XLSX)

S4 Table Descriptive statistics for the internal and external replication samples. (XLSX)

S5 Table Suggestive single-variant tests (minor allele frequencies ≥ 0.01) using ARIC participants with exome sequence. (XLSX)

S6 Table *ITPRIP* variants contributing to the T5 test of the aβ_42_:aβ_40_ ratio at visit 3 using ARIC participants with exome sequence. (XLSX)

S7 Table *PLIN2* variants contributing to the T5 test of the aβ_42_:aβ_40_ ratio at visit 3 using ARIC participants with exome sequence. (XLSX)

S8 Table *TSPAN18* variants contributing to the T5 test of the aβ_42_:aβ_40_ ratio at visit 3 using ARIC participants with exome sequence. (XLSX)

S9 Table *NCOA1* variants contributing to the SKAT test of the aβ_42_:aβ_40_ ratio at visit 3 using ARIC participants with exome sequence. (XLSX)

S10 Table *NT5C3B* variants contributing to the SKAT test of the fold-change in aβ_42_ using ARIC participants with exome sequence. (XLSX)

S11 Table Suggestive T5 gene-based associations (p-value < 5.98x10^-5^ and cumulative minor allele frequency≥0.5% in the suggestive analysis group) using ARIC participants with exome sequence. (XLSX)

S12 Table Suggestive SKAT associations (P-value < 5.98x10^-5^ and cumulative minor allele frequencies ≥0.5% in the suggestive analysis group) using ARIC participants with exome sequence. (XLSX)

S13 Table Functional variants (> 1 minor allele copy) overlapping in ARIC and FHS participants with exome sequence; contributors to the gene-based tests. (XLSX)

S14 Table Temporal sensitivity of significant single-variant tests in ARIC participants with exome sequence. (XLSX)

S15 Table Temporal sensitivity of significant gene-based tests in ARIC participants with exome sequence. (XLSX)

S16 Table Temporal sensitivity of suggestive single-variant results in ARIC participants with exome sequence. (XLSX)

S17 Table Temporal sensitivity of suggestive T5 tests in ARIC participants with exome sequence. (XLSX)

S18 Table Temporal sensitivity of suggestive SKAT tests in ARIC participants with exome sequence. (XLSX)

S19 Table Cross-trait results for significant single-variants in ARIC participants with exome sequence. (XLSX)

S20 Table Cross-trait results for significant genes in ARIC participants with exome sequence. (XLSX)

S21 Table Functions of significant genes identified by SKAT of ARIC African Americans with exome sequence. (XLSX)

S22 Table Sensitivity analysis for INNO-BIA plate effects; significant single-variant tests in ARIC participants with exome sequence. (XLSX)

S23 Table Sensitivity analysis for INNO-BIA plate effects: SKAT and T5 results using ARIC participants with exome sequence. (XLSX)

S24 Table *KLKB1* and *F12* single-variant tests stratified by *APOE* ε4 carrier status; ARIC participants with exome sequence. (XLSX)

S25 Table *KLKB1* and *F12* single-variant tests stratified by visit 5 cognitive status; ARIC participants with exome sequence. (XLSX)

S26 Table Significant T1 burden tests using ARIC participants with exome sequence. (XLSX)
