# Supplementary material for: Whole exome sequence-based association analyses of plasma amyloid-β in African and European Americans; the Atherosclerosis Risk in Communities-Neurocognitive Study
Source: PLoS One. 2017 Jul 13;12(7):e0180046. doi: 10.1371/journal.pone.0180046 (PMC5509141; doi:10.1371/journal.pone.0180046)
Supplement: S1 Fig — (PDF) [file pone.0180046.s001.pdf]

S1 Fig: QQ Plots For Single-variant Tests Using ARIC Participants With Exome Sequence

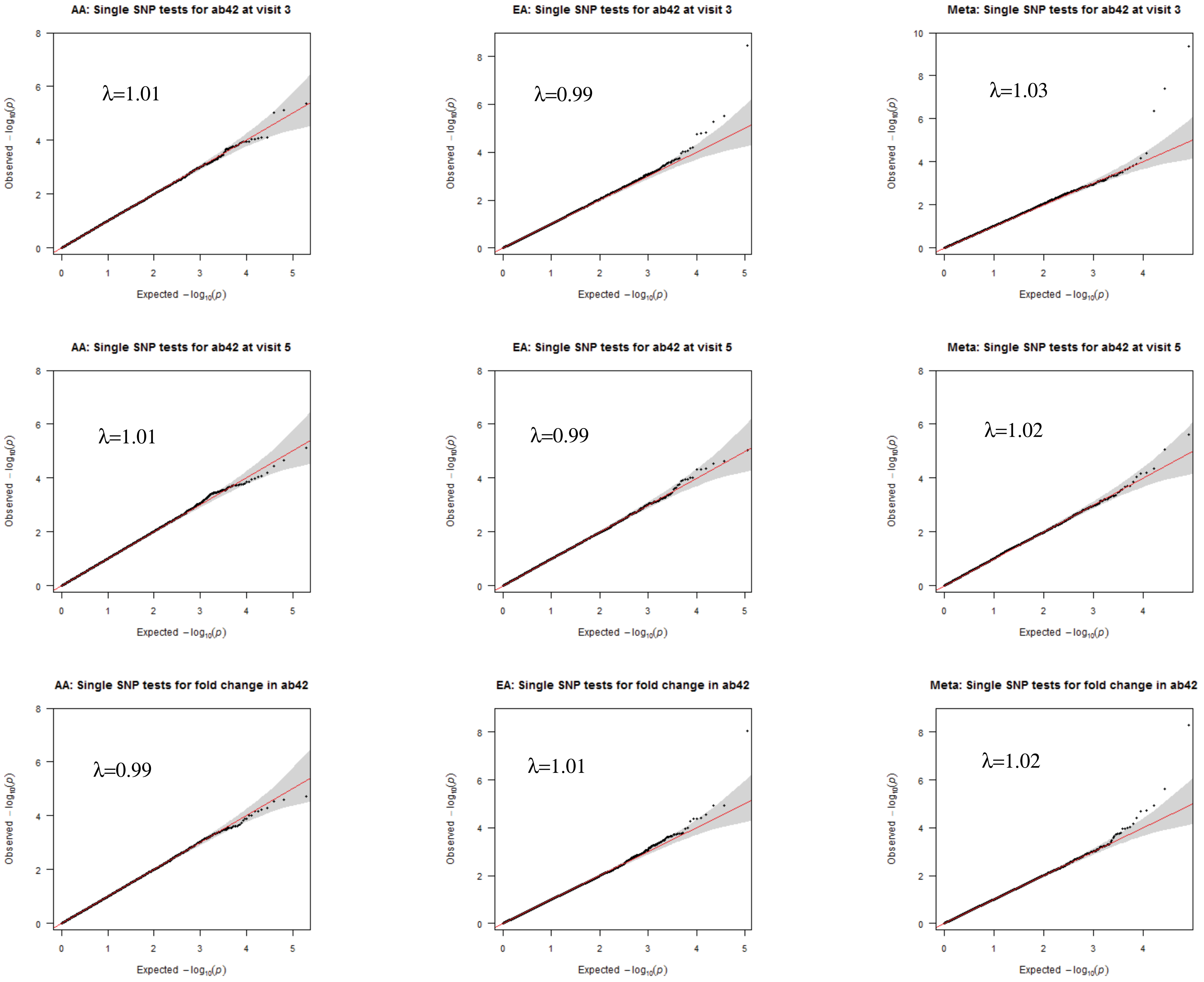

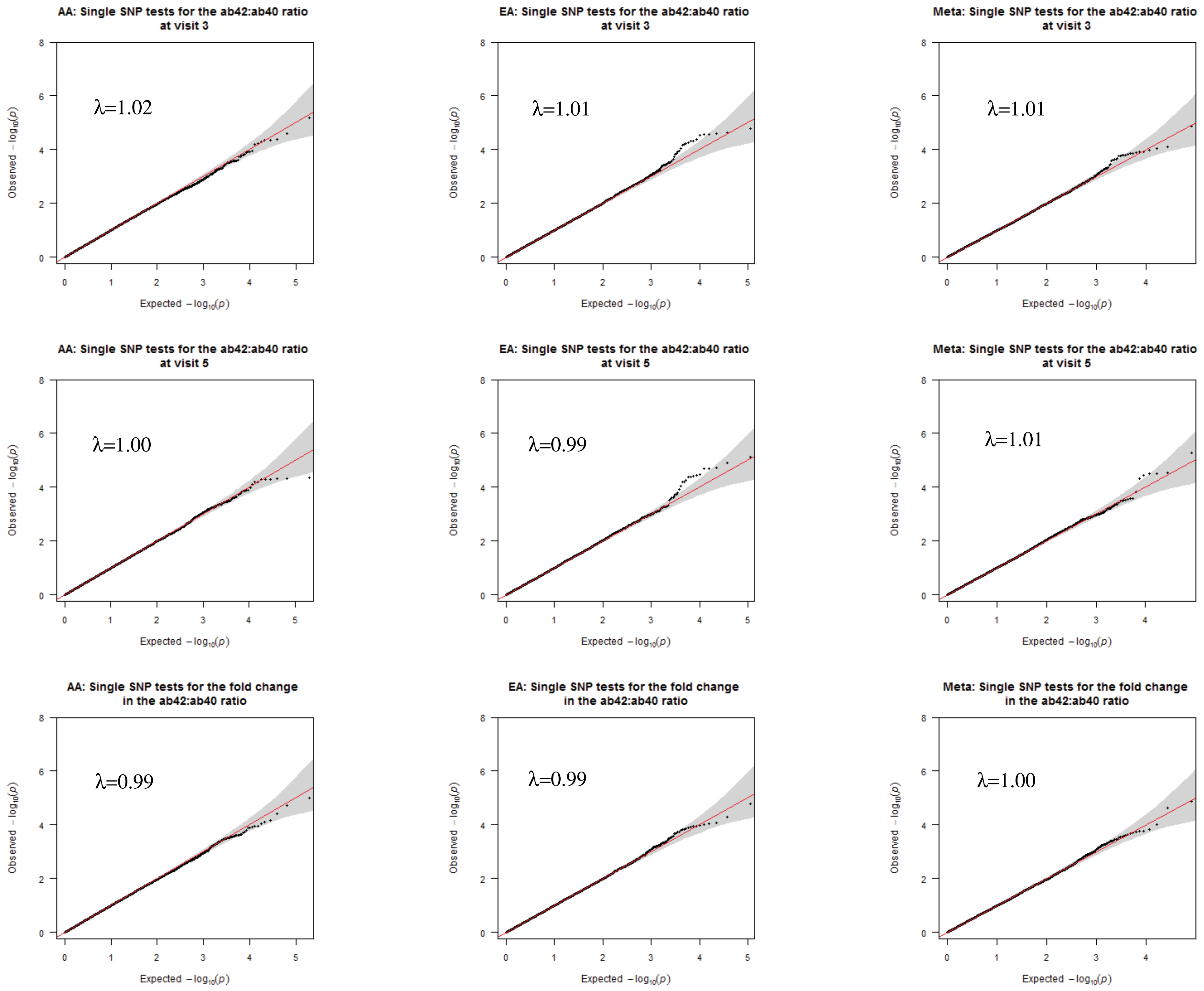
AA: Single SNP tests for the ab42:ab40 ratio at visit 3
λ=1.02
EA: Single SNP tests for the ab42:ab40 ratio at visit 3
λ=1.01
Meta: Single SNP tests for the ab42:ab40 ratio at visit 3
λ=1.01
AA: Single SNP tests for the ab42:ab40 ratio at visit 5
λ=1.00
EA: Single SNP tests for the ab42:ab40 ratio at visit 5
λ=0.99
Meta: Single SNP tests for the ab42:ab40 ratio at visit 5
λ=1.01
AA: Single SNP tests for the fold change in the ab42:ab40 ratio
λ=0.99
EA: Single SNP tests for the fold change in the ab42:ab40 ratio
λ=0.99
Meta: Single SNP tests for the fold change in the ab42:ab40 ratio
λ=1.00
Observed $-\log_{10}(p)$
Expected $-\log_{10}(p)$
