# Supplementary material for: Whole exome sequence-based association analyses of plasma amyloid-β in African and European Americans; the Atherosclerosis Risk in Communities-Neurocognitive Study
Source: PLoS One. 2017 Jul 13;12(7):e0180046. doi: 10.1371/journal.pone.0180046 (PMC5509141; doi:10.1371/journal.pone.0180046)
Supplement: S2 Fig — (PDF) [file pone.0180046.s002.pdf]

S2 Fig: QQ Plots For T5 Burden Tests Using ARIC Participants With Exome Sequence

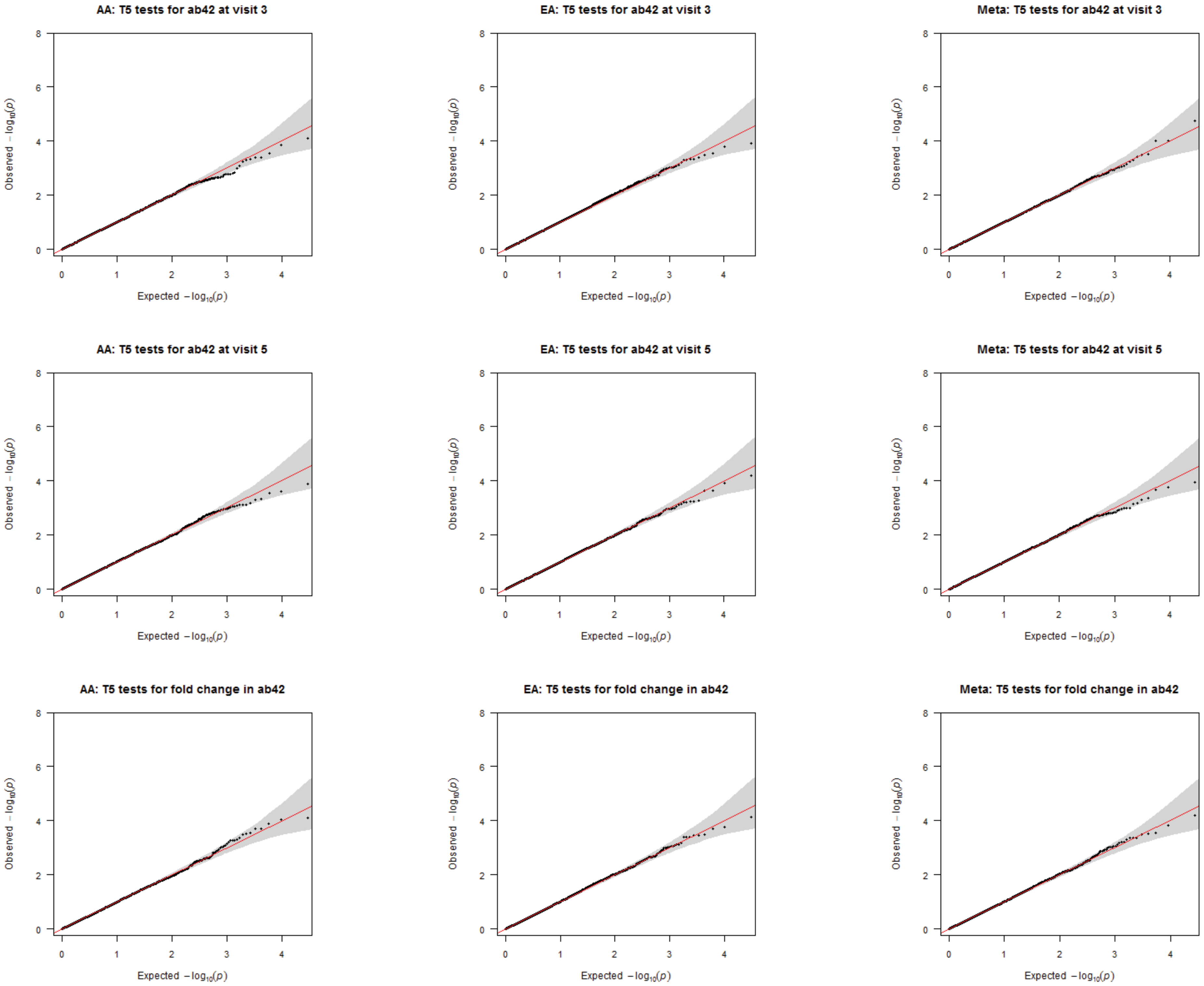

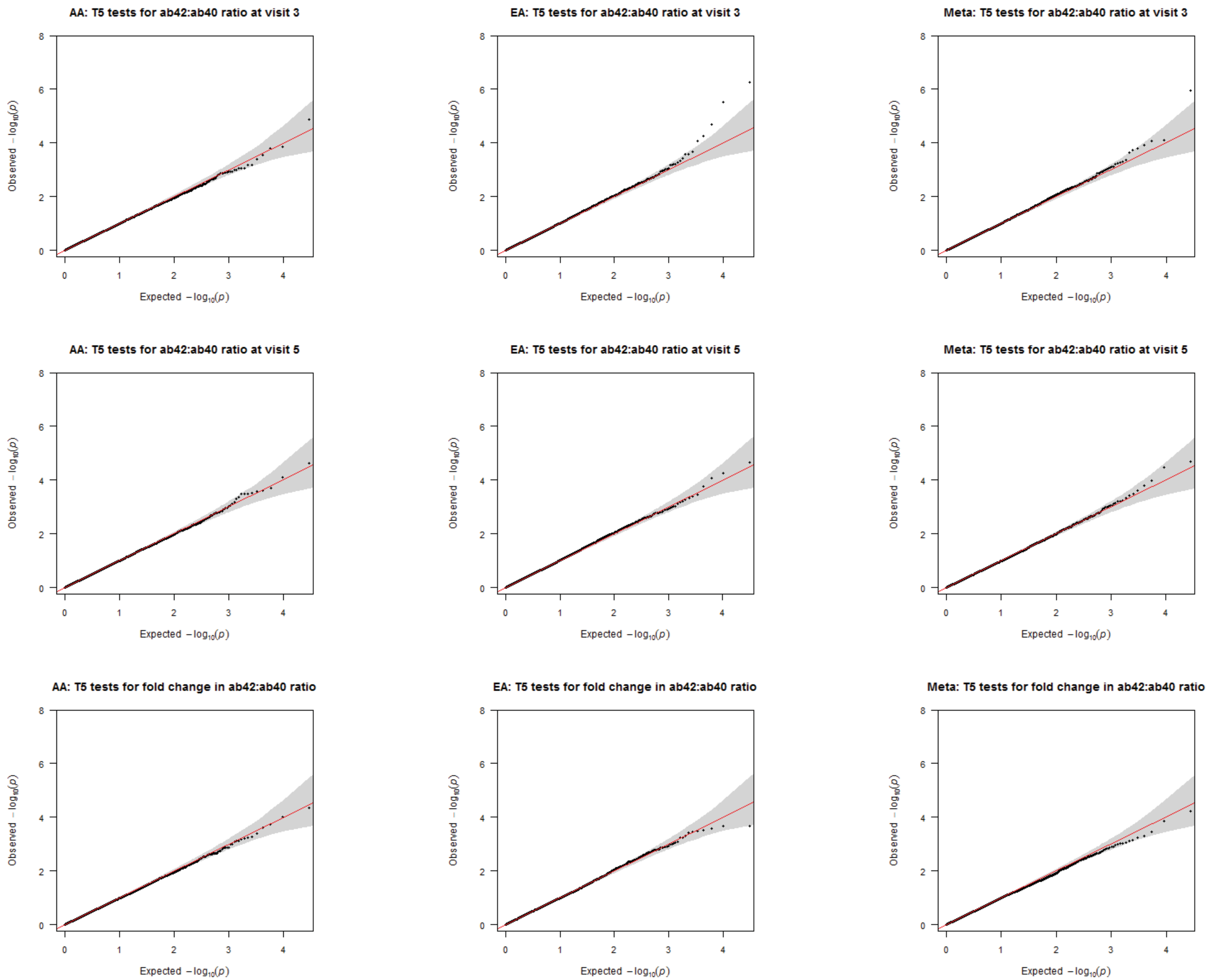
AA: T5 tests for ab42:ab40 ratio at visit 3
EA: T5 tests for ab42:ab40 ratio at visit 3
Meta: T5 tests for ab42:ab40 ratio at visit 3
AA: T5 tests for ab42:ab40 ratio at visit 5
EA: T5 tests for ab42:ab40 ratio at visit 5
Meta: T5 tests for ab42:ab40 ratio at visit 5
AA: T5 tests for fold change in ab42:ab40 ratio
EA: T5 tests for fold change in ab42:ab40 ratio
Meta: T5 tests for fold change in ab42:ab40 ratio
Expected $-\log_{10}(p)$
Observed $-\log_{10}(p)$
