# Supplementary material for: Whole exome sequence-based association analyses of plasma amyloid-β in African and European Americans; the Atherosclerosis Risk in Communities-Neurocognitive Study
Source: PLoS One. 2017 Jul 13;12(7):e0180046. doi: 10.1371/journal.pone.0180046 (PMC5509141; doi:10.1371/journal.pone.0180046)
Supplement: S5 Fig — (PDF) [file pone.0180046.s005.pdf]

S5 Fig: QQ Plots for the T5 Test of the Third Visit aβ_{42}:aβ_{40} Ratio from the Cross-Race Meta-analysis Using Different Minor Allele Count Thresholds (Starting at 0.5% CMAF)

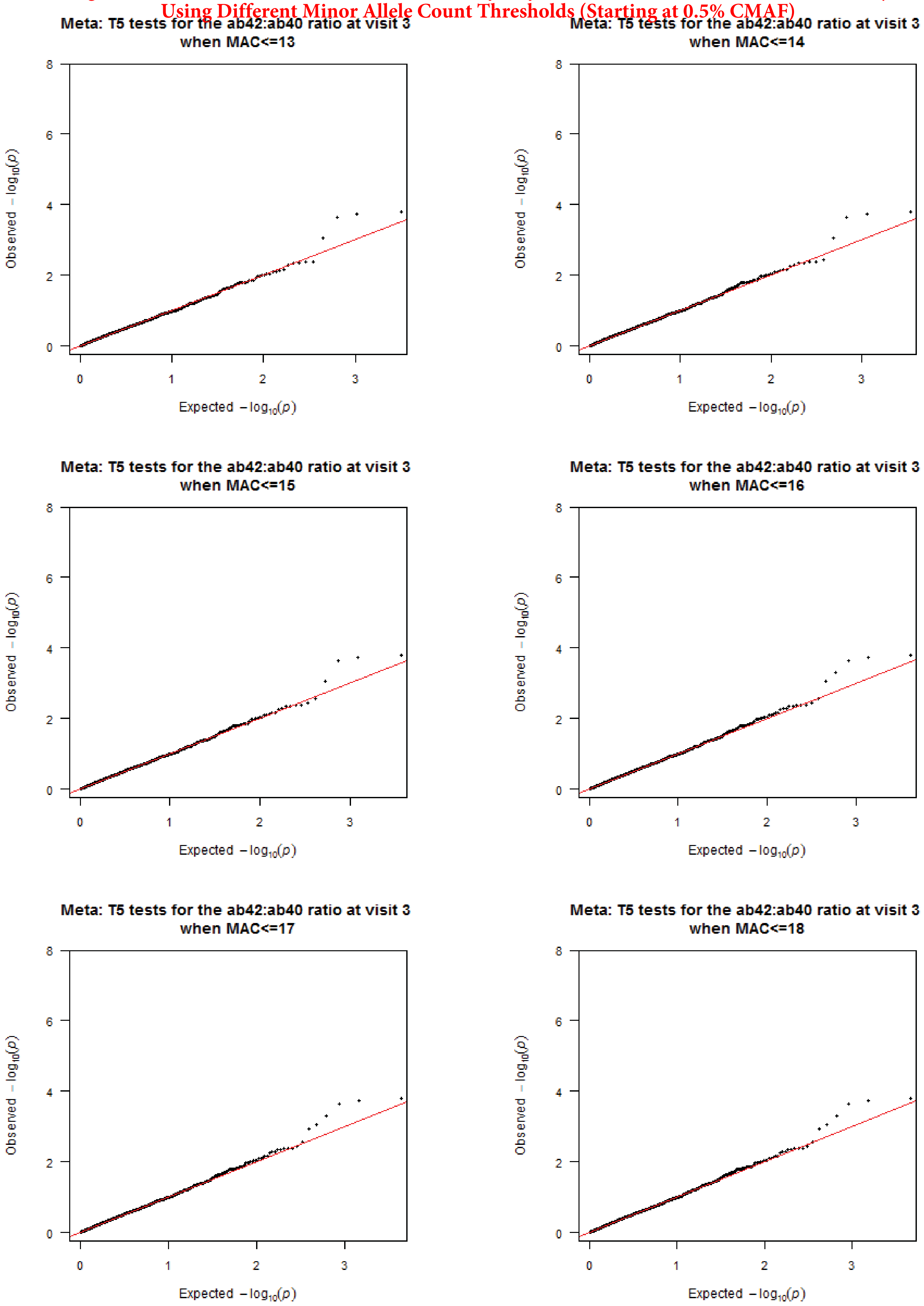

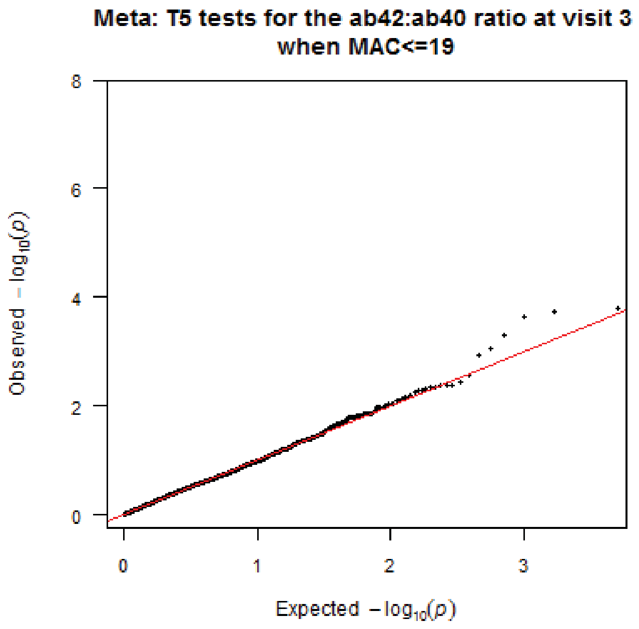
Meta: T5 tests for the ab42:ab40 ratio at visit 3
when MAC<=19
Observed $-\log_{10}(p)$
Expected $-\log_{10}(p)$
0
2
4
6
8
0
1
2
3

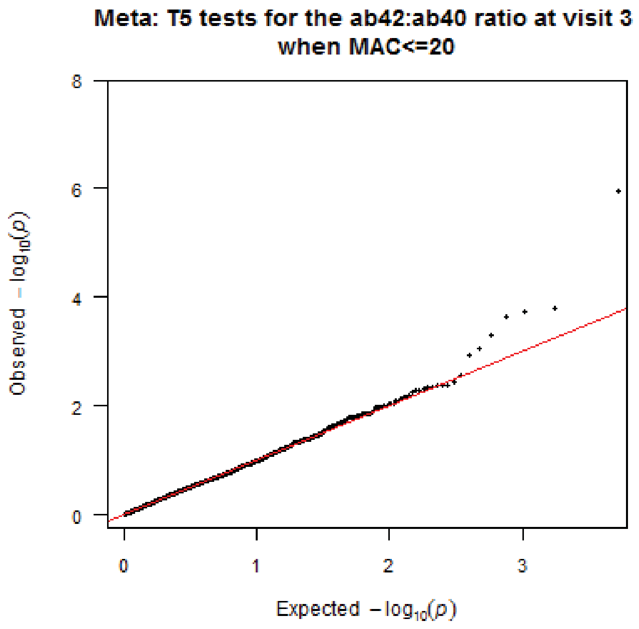
Meta: T5 tests for the ab42:ab40 ratio at visit 3
when MAC<=20
Observed $-\log_{10}(p)$
Expected $-\log_{10}(p)$
0
2
4
6
8
0
1
2
3
