# Supplementary material for: Whole exome sequence-based association analyses of plasma amyloid-β in African and European Americans; the Atherosclerosis Risk in Communities-Neurocognitive Study
Source: PLoS One. 2017 Jul 13;12(7):e0180046. doi: 10.1371/journal.pone.0180046 (PMC5509141; doi:10.1371/journal.pone.0180046)

S6 Fig: QQ Plots for the SKAT Test of the Third Visit aβ_{42}:aβ_{40} Ratio in AAs Using Different Minor Allele Count Thresholds (Starting at 0.5% CMAF)

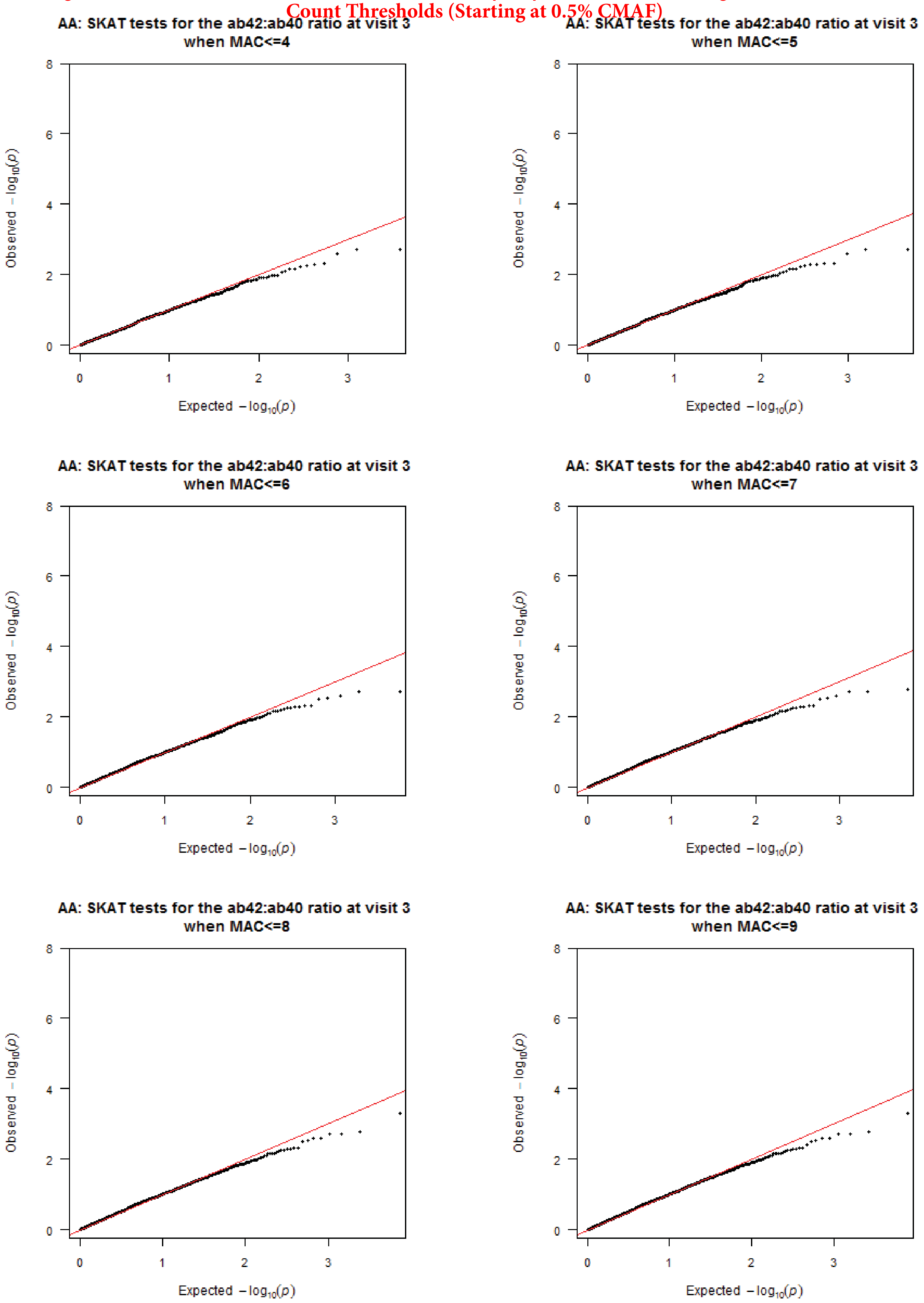

Supplement: S6 Fig — (PDF) [file pone.0180046.s006.pdf]
